# Physiological, Metabolic, and Transcriptomic Analyses Reveal Mechanisms of Proliferation and Somatic Embryogenesis of Litchi (*Litchi chinensis* Sonn.) Embryogenic Callus Promoted by D-Arginine Treatment

**DOI:** 10.3390/ijms25073965

**Published:** 2024-04-02

**Authors:** Ludan Cao, Guo Wang, Xiuxu Ye, Fang Li, Shujun Wang, Huanling Li, Peng Wang, Jiabao Wang

**Affiliations:** 1 School of Tropical Agriculture and Forestry, Hainan University, Haikou 570228, China; 21220951310141@hainanu.edu.cn; 2 Environment and Plant Protection Institute, Chinese Academy of Tropical Agricultural Sciences, Haikou 571101, China; wanglucai@sina.com (G.W.); lifang200709@126.com (F.L.); wshujun86@163.com (S.W.); 115-10@163.com (H.L.); 3 Tropical Crops Genetic Resources Institute, Chinese Academy of Tropical Agricultural Sciences, Haikou 571101, China; yxx740234283@sina.cn

**Keywords:** D-Arg, litchi, polyamine metabolism, plant endogenous hormones, transcriptome analysis, embryogenic callus, somatic embryogenesis

## Abstract

D-arginine (D-Arg) can promote embryogenic callus (EC) proliferation and increase the rate of somatic embryo induction of litchi (*Litchi chinensis* Sonn.), yet the mechanism underlying the processes is incompletely understood. To investigate the mechanism, physiological responses of polyamines (PAs) [putrescine (Put), spermidine (Spd), and spermine (Spm)] were investigated for D-Arg-treated litchi EC and enzyme activity related to polyamine metabolism, plant endogenous hormones, and polyamine- and embryogenic-related genes were explored. Results showed that the exogenous addition of D-Arg reduces the activity of diamine oxidase (DAO) and polyamine oxidase (PAO) in EC, reduces the production of H_2_O_2_, promotes EC proliferation, and increases the (Spd + Spm)/Put ratio to promote somatic embryo induction. Exogenous D-Arg application promoted somatic embryogenesis (SE) by increasing indole-3-acetyl glycine (IAA-Gly), kinetin-9-glucoside (K9G), and dihydrozeatin-7-glucoside (DHZ7G) levels and decreasing trans-zeatin riboside (tZR), N-[(-)-jasmonoyl]-(L)-valine (JA-Val), jasmonic acid (JA), and jasmonoyl-L-isoleucine (Ja-ILE) levels on 18 d, as well as promoting cell division and differentiation. The application of exogenous D-Arg regulated EC proliferation and somatic embryo induction by altering gene expression levels of the WRKY family, AP2/ERF family, C3H family, and C2H2 family. These results indicate that exogenous D-Arg could regulate the proliferation of EC and the SE induction of litchi by changing the biosynthesis of PAs through the alteration of gene expression pattern and endogenous hormone metabolism.

## 1. Introduction

Polyamines (PAs) are nitrogenous substances widely distributed in plants [1]. PAs mainly include putrescine (Put), spermidine (Spd), and spermine (Spm) [2]. These PA components regulate various physiological activities, such as embryogenesis, organogenesis, flower generation, fruit ripening, and senescence [1,2]. Polyamine metabolism has been extensively investigated in higher plants [1,3]. Put is the central product of the polyamine synthesis pathway and the biosynthetic precursor of Spd and Spm [4,5]. It can be synthesized by arginine decarboxylase (ADC)-catalyzed reaction and ornithine decarboxylase (ODC)-catalyzed reaction [6]. Spd and Spm are formed by the sequential addition of aminopropyl groups provided by decarboxylated S-adenosylmethionine (dcSAM) to Put and Spd by spermidine synthase (SPDS) and spermine synthase (SPMS), respectively [7]. The dcSAM is formed by the decarboxylation reaction of S-adenosylmethionine (SAM) through S-adenosylmethionine decarboxylase (SAMDC) [5]. The oxidation of PAs is mainly carried out by diamine oxidase (DAO) and polyamine oxidase (PAO), which produces H_2_O_2_ through oxidation [8]. D-arginine (D-Arg) is an ADC inhibitor, which can inhibit the synthesis of Put and affect the biosynthesis of endogenous PAs by reducing the activity of ADC [9]. In soybeans, D-Arg exacerbates the root damage caused by sodium chloride by altering PAs content [10]. In cotton, exogenous D-Arg can significantly reduce endogenous Put levels, thereby lowering PAs level [11]. In lettuce, the application of D-Arg can reduce endogenous PAs level and reduce the tolerance of lettuce seedlings to high temperatures [12].

Somatic embryogenesis (SE) via embryogenic callus (EC) is an essential mode of regeneration [13] and provides great potential for in vitro culture efficiency [14,15]. The ability to initiate embryogenic cultures is controlled by many factors, including endogenous genotype-specific factors, source of the explants, culture medium formula (sugar, plant growth regulator, activated charcoal, and glycerol), and physical environments (light, temperature, and osmotic stress) [15,16]. It has been proven that PAs play a crucial role in the development of SE [1,17,18]. Montague et al. demonstrated that PA content in carrot cells was related to SE [19]. Longan embryo development was reported to be promoted by higher (Spd + Spm)/Put [20]. A high proportion of Put/(Spd + Spm) is conducive to inducing the generation of Scots pine EC [18]. It was reported that exogenous PAs or polyamine inhibitors can regulate the process of SE [21]. Exogenous D-Arg significantly inhibits the callus proliferation of four cotton varieties, while exogenous Put promoted callus proliferation and somatic embryo differentiation to some extent [22]. Exogenous D-Arg can inhibit the transformation of longan EC to globular embryos (GE) [2].

Litchi (*Litchi chinensis* Sonn.) is an important subtropical fruit that is valued for its unique fruit flavor, exquisite sweetness, and luscious juiciness [23]. Litchi breeding plays a crucial role in enhancing the quality, productivity, and disease resistance of this beloved fruit, ensuring its sustainability and delighting consumers worldwide [23]. SE is a method that has multiple applications in plant improvement. It is the basis of creating new germplasm via biotechnologies, such as gene editing and transgene, and greatly shortens the breeding period [24,25]. Our previous studies showed that the application of D-Arg to callus proliferation culture medium could promote EC proliferation and improve the somatic embryo induction of ‘Feizixiao’ litchi [26]. However, the underlying mechanism of these processes is not fully understood yet.

In this article, the effects of exogenous D-Arg on EC proliferation and somatic embryo induction are investigated. The changes of PAs, polyamine metabolism-related enzyme activity, plant endogenous hormones, and deferentially expressed genes in EC at certain key stages cultured on the medium with or without D-Arg were tested. The results could provide a basis for further exploring the mechanism of exogenous D-Arg on EC proliferation and somatic embryo induction in litchi.

## 2. Results

### 2.1. Effect of Exogenous D-Arg Application on the Proliferation of EC and Somatic Embryo Induction of Litchi

**Figure 1:**
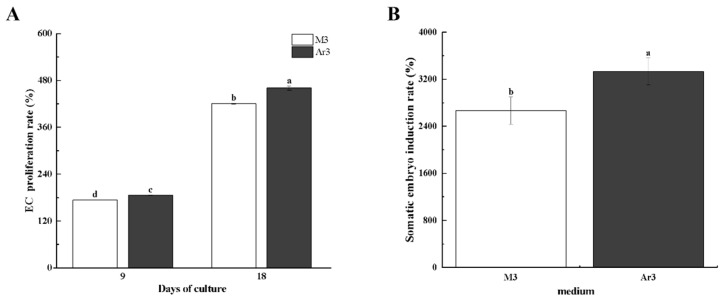
Effect of D-Arg on proliferation of EC (**A**) and somatic embryo induction rate (**B**) of litchi. Data were presented as means (±SE) from three independent biological replicates. Different letters indicate significant differences among treatments at *p* < 0.05 level.

We first tested the role of D-Arg in EC proliferation and somatic embryo induction for litchi. Two medium formulas were used, with M3 as the control without D-Arg and Ar3 as the treatment with 0.35 g·L^−1^ (2 mmol·L^−1^) D-Arg included. As shown in Figure 1A, the EC proliferation rate was significantly higher in the Ar3 medium than in the M3 medium (by approximately 7–10%), suggesting a positive role of D-Arg in promoting litchi EC proliferation (Figure 1A). Exogenous D-Arg application could also increase the SE, since the number of somatic embryos induced from EC cultured on Ar3 medium is significantly higher (by 25%) than that of callus cultured on M3 medium (Figure 1B).

### 2.2. Effect of Exogenous D-Arg Application on PA Metabolism in Litchi EC

**Figure 2:**
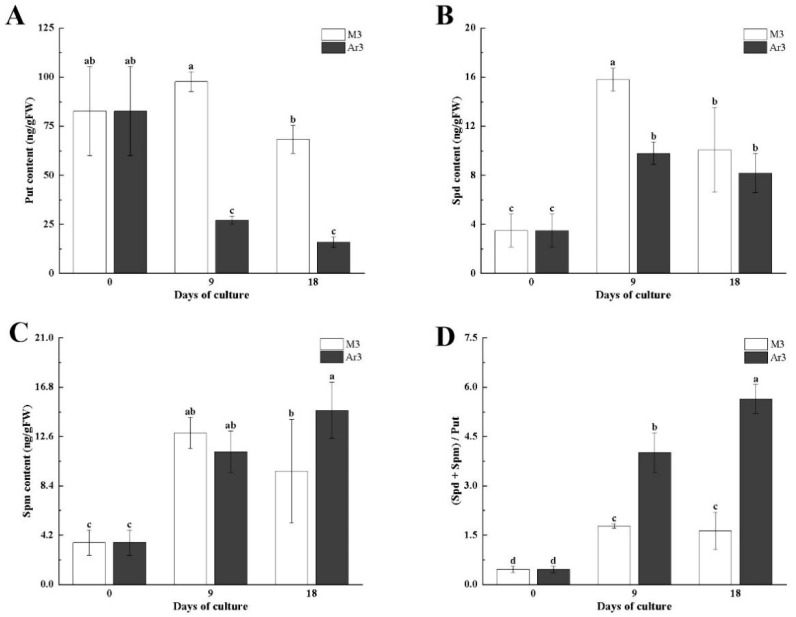
Effect of D-Arg on PAs content in litchi EC: (**A**) Put content; (**B**) Spd content; (**C**) Spm content; (**D**) (Spd + Spm)/Put. Data were presented as means (±SE) from three independent biological replicates. Different letters indicate significant differences among treatments at *p* < 0.05 level.

To investigate the metabolic effect of D-Arg application, PAs were measured in litchi EC. The Put content in EC cultured on M3 medium was significantly decreased on 18 d compared with 9 d (by 30%) while the Put content in EC cultured on Ar3 medium was significantly decreased on 9 d (by 67%). Both at 9 d and 18 d, the contents of Put in EC on the Ar3 medium were significantly lower than that on the M3 medium, by approximately 72–76% (Figure 2A). The Spd content in both the control and treatment groups increased significantly (by 100%) at 9 d. Only at 9 d, the Spd content of callus on the M3 medium was significantly higher than that on the Ar3 medium (by 40%), and there was no significant difference between them at 18 d (Figure 2B). When EC was cultured at M3 and Ar3 media until 9 d, spermine content significantly increased (by 100%). There was no significant difference in Spm content between 18 d and 9 d of cultivation on both mediums. However, at 18 d, the spermine content of callus tissue on the Ar3 medium was significantly higher (by 55%) than in the control callus (Figure 2C). The (Spd + Spm)/Put ratio in callus tissue on both media significantly increased at 9 d (by 100%), while at 18 d, it was significantly higher in the Ar3 medium than at 9 d (by 40%), but there was no significant difference in the M3 medium. At both 9 d and 18 d, the ratio of (Spd + Spm)/Put in EC on the Ar3 medium was significantly higher (by 100%) than in the M3 medium (Figure 2D). The results indicate that exogenous D-Arg affected the endogenous polyamine levels of litchi EC (Figure 2).

**Figure 3:**
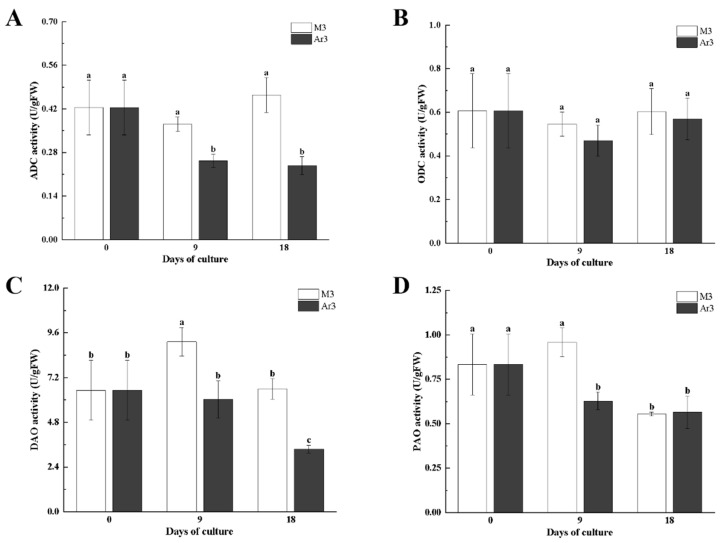
Effect of D-Arg on the activities of polyamine metabolism in litchi EC: (**A**) ADC activity; (**B**) ODC activity; (**C**) DAO activity; (**D**) PAO activity. Data were presented as means (±SE) from three independent biological replicates. Different letters indicate significant differences among treatments at *p* < 0.05 level.

Furthermore, the activities of polyamine synthase and oxidase enzymes were measured in litchi EC. ADC activity in EC cultured in Ar3 medium decreased significantly at 9 d (by 12%), but there was no significant change of ADC activity in EC cultured in M3 medium. ADC activity in EC on the Ar3 medium was significantly lower (by 32–48%) than on the M3 medium at both 9 d and 18 d (Figure 3A). There was no significant difference in ODC activity between the control group and the treatment group (Figure 3B). The results indicated that exogenous D-Arg only affects the arginine pathway and not the ornithine pathway. The DAO activity of EC was significantly increased when cultured in M3 to 9 d (by 40%), then decreased significantly on 18 d (by 27%). However, the DAO activity of EC on the Ar3 medium decreased significantly at 18 d (by 50%) (Figure 3C). DAO activity in EC on the Ar3 medium was 34.3–48% lower than on the M3 medium at both 9 d and 18 d (Figure 3C). The PAO activity of EC on the M3 medium decreased significantly at 18 d (by approximately 42%), but that happened at 9 d on the Ar3 medium (by 24%). PAO activity was significantly higher in the control group (by 34%) than in the treatment group only on 9 d, and there was no significant difference between the two groups at 18 d (Table S3, Figure 3D). The results show that exogenous D-Arg significantly inhibited the activity of catabolizing enzymes in polyamine metabolism.

### 2.3. Effect of Exogenous D-Arg Application on Endogenous Hormone Level in Litchi

**Figure 4:**
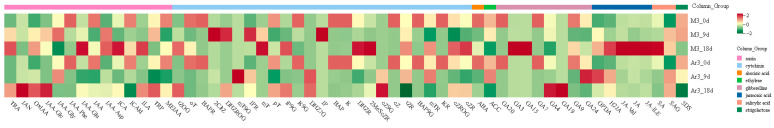
Effect of exogenous D-Arg on endogenous hormone levels in litchi EC. Data were presented as means from three independent biological replicates. Perform column standardization during data visualization.

To test how enzyme activities related to PA metabolism were regulated, hormone levels in litchi EC were measured (Figure 4). A total of 58 molecules of hormones were detected, including 14 auxins, 26 cytokinins, one abscisic acid, one ethylene, eight gibberellins, five jasmonic acids, two salicylic acids, and one aurolactone. Using VIP > 1, |log2Fold Change| ≥ 1 and *p*-value < 0.05 as standard for screening differential metabolites, there were no differential metabolites in EC subcultured on M3 and Ar3 medium at 9 d, indicating that the application of D-Arg did not cause changes in endogenous hormone levels in litchi callus at 9 d. The differential metabolites of M3_18 d vs. Ar3_18 d include indole-3-acetyl glcine (IAA-Gly), kinetin-9-glucoside (K9G), dihydrozeatin-7-glucoside (DHZ7G), trans-zeatin riboside (tZR), N-[(-)-jasmonoyl]-(L)-valine (JA-Val), jasmonic acid (JA), and jasmonoyl-L-isoleucine (JA-ILE). Among them, Exogenous D-Arg can increase the expression level of IAA-Gly by 100%, K9G by 100%, and DHZ7G by 10%, and can decrease the expression level of tZR by 75%, JA-VAL by 88%, JA by 82%, and JA-ILE by 87%. The changes of these differential metabolites during culture were as follows: the IAA-Gly content of EC on the M3 medium decreased significantly at 18 d, while the IAA-Gly content of EC on the Ar3 medium showed no significant difference during culture. The K9G content of EC on M3 medium and Ar3 medium showed the same change trend and both decreased significantly on 9 d. There was no significant change in DHZ7G content in the control group, but the DHZ7G content in the treatment group increased significantly at 18 d. The tZR content of EC on the two media did not change significantly during the culture process. The changing trend of JA-Val and JA-ILE was the same, and the content of JA-Val and JA-ILE increased significantly at 18 d of the M3 medium; there was no significant difference in the process of the Ar3 medium. The JA content of EC on the M3 medium decreased significantly at 9 d and increased significantly at 18 d compared with that at 9 d, while there was no significant difference in content on the Ar3 medium.

### 2.4. Investigation of DEGs and Functional Categorization

**Figure 5:**
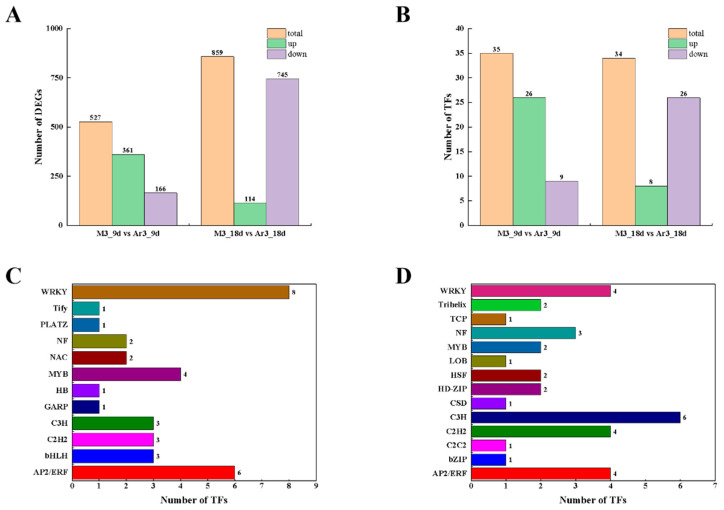
Analysis of DEGs and TFs: (**A**) the number of DEGs up- or down-regulated in the different comparisons; (**B**) the number of differentially expressed TFs up- or down-regulated in the different comparisons; (**C**) classification of TFs assigned of M3_9 d vs. Ar3_9 d group; (**D**) classification of TFs assigned of M3_18 d vs. Ar3_18 d group.

The number of DEGs in the M3_9 d vs. Ar3_9 d group and M3_18 d vs. Ar3_18 d group were 527 and 859, respectively (Figure 5A). In the M3_9 d vs. Ar3_9 d group, 361 genes were significantly upregulated and 166 were significantly downregulated. In the M3_18 d vs. Ar3_18 d group, 114 genes were significantly upregulated and 745 were significantly downregulated.

**Figure 6:**
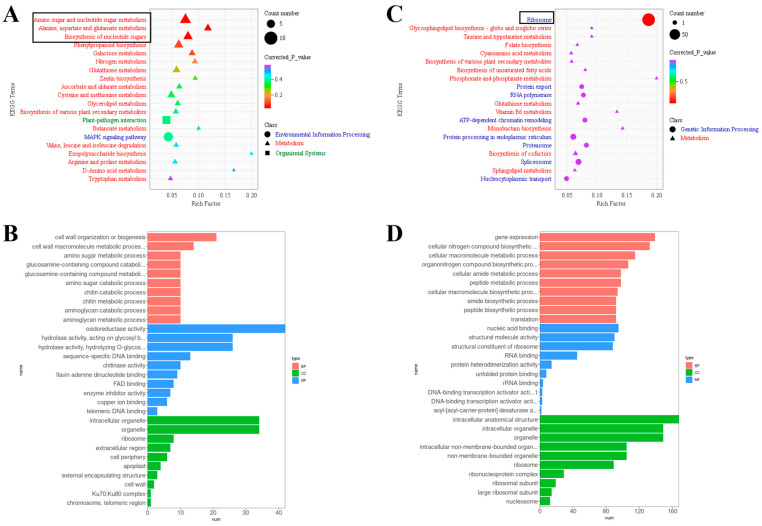
KEGG and GO enrichment analysis of the differential genes: (**A**) KEGG enrichment of differential genes at M3_9 d vs. Ar3_9 d group; (**B**) KEGG enrichment of differential genes at M3_9 d vs. Ar3_9 d group; (**C**) GO enrichment of differential genes at M3_18 d vs. Ar3_18 d group; (**D**) GO enrichment of differential genes at M3_18 d vs. Ar3_18 d group. The black box represents significantly enriched metabolic pathways (*p* < 0.05).

The DEGs of the M3_9 d vs. Ar3_9 d group and M3_18 d vs. Ar3_18 d group were annotated with the KEGG database. “Amino sugar and nucleotide sugar metabolism”, “alanine, aspartate, and glutamate metabolism”, and “biosynthesis of nucleotide sugars” were significantly enriched in the M3_9 d vs. Ar3_9 d group (Figure 6A). In the M3_18 d vs. Ar3_18 d group, “ribosome” was significantly enriched (Figure 6C).

To further reveal their functions, the DEGs of the M3_9 d vs. Ar3_9 d group and the M3_18 d vs. Ar3_18 d group were annotated with the GO database. In the M3_9 d vs. Ar3_9 d group, in the biological process category, the majority of genes were annotated as “cell wall organization or biogenesis” and “cell wall macromolecule metabolic process”. Within the molecular function category, the highly represented GO terms included “oxidoreductase activity”, “hydrolase activity, acting on glycosyl bonds”, and “hydrolase activity, hydrolyzing O-glycosyl compounds”, while “intracellular organelle”, “organelle”, and “ribosome” were highly represented in the cellular component category (Figure 6B). In the M3_18 d vs. Ar3_18 d group, within the biological process category, the highly represented GO terms included “gene expression”, “cellular nitrogen compound biosynthetic process”, and “cellular macromolecule metabolic process”, while “nucleic acid binding”, “RNA binding”, and “structural molecule activity” were highly represented in the molecular function category. For the cellular component category, the majority of genes were annotated with “intracellular anatomical structure”, “intracellular organelle”, and “organelle” (Figure 6D).

**Figure 7:**
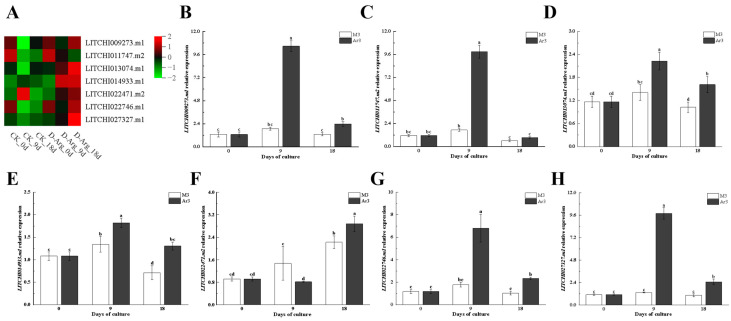
Expression of genes in litchi EC treated with exogenous D-Arg by RNA-seq (**A**) and qRT-PCR (**B**–**H**). Different letters indicate significant differences among treatments at *p* < 0.05 level.

Through qRT-PCR analysis, seven DEGs were randomly selected to validate the transcriptome data. As shown in Figure 7, the relative expression levels of the selected gene treatment group were consistent with the transcriptional changes compared to the control group (Figure 7).

### 2.5. Differentially Expressed TFs

In the M3_9 d vs. Ar3_9 d group, 35 TFs were differentially expressed with 26 being up-regulated and nine being down-regulated (Figure 5B). These included eight WRKY family genes (all up-regulated), six AP2/ERF family genes (five up-regulated), three C3H family genes, three C2H2 family genes, and three bHLH family genes (Table S1, Figure 5C). In the M3_18 d vs. Ar3_18 d group, there were 34 differentially expressed, TFs of which eight were up-regulated and 26 were down-regulated (Figure 5B). The predominant TF families included C3H with six genes (three up-regulated), followed by four C2H2 genes (one up-regulated), four WRKY family genes (three up-regulated), and four AP2/ERF family genes (all down-regulated) (Table S2, Figure 5D).

### 2.6. Identification of Genes Related to PA Metabolism

The genes involved in the metabolism of PAs in *Arabidopsis thaliana* and tomato correspond to seven gene families (ODC, ADC, SAMDC, SPDS, SPMS, DAO, and PAO). The protein sequences of these gene families were analyzed through the Pfam database to identify the conserved domain of each gene family (Table 1). According to the above conserved domains, 44 genes involved in PA metabolism were identified in the litchi genome, which included two members of the ODC gene family, one member of the ADC gene family, three members of the SAMDC gene family, eight members of the SPDS/SPMS gene family, seven members of the DAO gene family, and 23 members of the PAO gene family (Table 1).

### 2.7. Expression Analysis of PA Metabolism Genes from the Litchi Genome in EC

**Figure 8:**
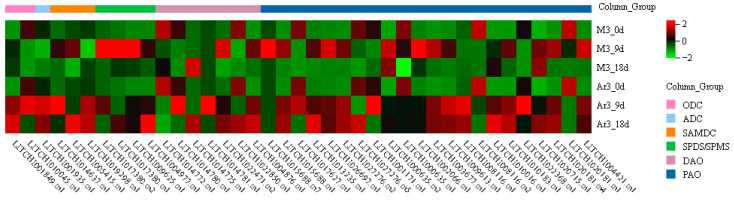
Expression (TPM) of PA metabolism gene in litchi EC treated with exogenous D-Arg.

A heat map was drawn according to the TPM values of the genes involved in PA metabolism (Figure 8). *LITCHI019019.m1*, *LITCHI014771.m1*, *LITCHI014771.m5*, *LITCHI009625.m3*, and *LITCHI022355.m1* were not expressed in litchi callus and were hence deleted without further analysis (Table 1). D-Arg treatment significantly increased the expression levels of *LITCHI010045.m1* (ODC family), *LITCHI001935.m1* (ADC family), *LITCHI014780.m1* (DAO family), *LITCHI022850.m1* (DAO family), *LITCHI017627.m1* (PAO family), *LITCHI009613.m1* (PAO family), and *LITCHI008116.m1* (PAO family) at both incubation times. In addition, at 9 d, D-Arg treatment significantly upregulated the expression levels of *LITCHI014637.m1* (SAMDC family) and *LITCHI001171.m1* (PAO family), and significantly downregulated the expression levels of *LITCHI017380.m1* (SPDS/SPMS family), *LITCHI017380.m2* (SPDS/SPMS family), *LITCHI000635.m2* (PAO family), and *LITCHI002066.m1* (PAO family). At 18 d, most genes of the polyamine metabolism gene family were up-regulated with D-Arg treatment.

**Figure 9:**
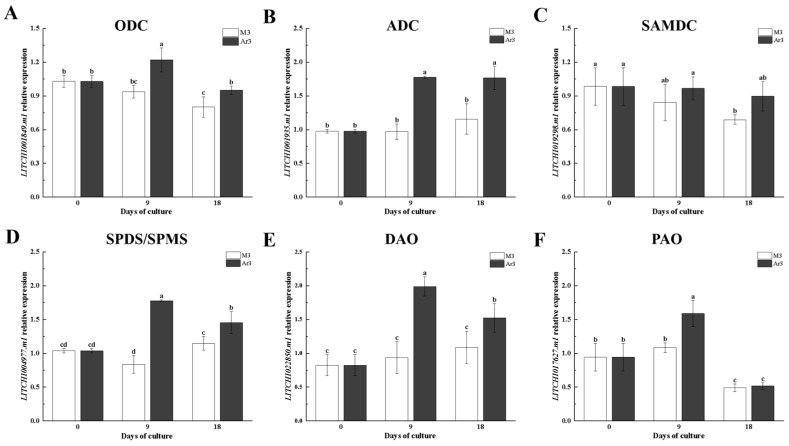
Expression levels of genes related to PA biosynthesis (**A**–**D**) and catabolism (**E**,**F**) in litchi EC proliferation with exogenous D-Arg by qRT-PCR. Different letters indicate significant differences among treatments at *p* < 0.05 level.

One gene from each PA gene family was randomly selected respectively to verify their expression by qRT-PCR. The results showed that exogenous D-Arg altered the expression levels of multiple genes related to polyamine synthesis and catabolic enzymes (Figure 9). The results indicate that the application of exogenous D-Arg affects the expression of several genes related to PA metabolism (Figure 8 and Figure 9).

## 3. Discussion

### 3.1. Exogenous D-Arg Treatment Regulates EC Proliferation and Somatic Embryo Induction in Litchi by Changing PA Levels

Different levels of D-Arg have different effects on SE in different plants. Exogenous 1 mmol·L^−1^ D-Arg could significantly inhibit the proliferation of cotton callus [22]. 0.01 mmol·L^−1^ of D-Arg could inhibit the transformation of EC into GE in longan [2]. Our group has previously studied the effects of exogenous addition of 0.5, 1, 2, and 4 mmol·L^−1^ D-Arg on EC proliferation and somatic embryo induction of litchi, and the results showed that exogenous addition of 2 mmol·L^−1^ had the greatest effect on EC proliferation and somatic embryo induction of litchi [26]. In this study, the results are consistent with the previous research results of our research group; the administration of 2 mmol·L^−1^ (0.35 g·L^−1^) D-Arg significantly increased EC proliferation and SE induction rates compared to the control (Figure 1).

Exogenous PAs and PA synthesis inhibitors have been widely used to study the role of PAs in plant SE [20]. D-Arg is an inhibitor of the polyamine biosynthesis pathway [9]. The content of Put is the most abundant among PAs, so the level of PAs mainly depends on Put consistent previous results [14]. In this study, exogenous D-Arg application significantly attenuated Put content, ADC activity, and DAO activity, while ODC activity was not significantly different from the control (Figure 2 and Figure 3). The Spd content and PAO activity of D-Arg treated EC were significantly lower than that in the control at 9 d, but the Spd content and PAO activity were not different from that in the control at 18 d (Figure 2 and Figure 3). The EC cultured with D-Arg treatment showed no significant difference in Spm content compared to the control at 9 d, but the Spm content was significantly higher than that of the control at 18 d (Figure 2 and Figure 3). The research results of Hashem in Arabidopsis are consistent with the results of this study for 18 days, exogenous 0.1 mmol D-Arg reduced Put, promoted Spm, and had no effect on Spd [27]. These results indicated that the synthesis of Put was mainly through arginine synthesis in litchi EC. At 9 d, exogenous D-Arg acted as an ADC inhibitor, which inhibited the ADC activity, reduced the synthesis of Put, lowered DAO activity that catalyzes Put decomposition, and lowered Spd synthesis; it had no significant effect on Spm, inhibiting PAO activity. The decrease in DAO and PAO activity will reduce the production of H_2_O_2_, prevent membrane lipid peroxidation, reduce cell wall hardening, facilitate cell division, and promote EC proliferation [28]. The factors that affect the content of Spd and Spm in the polyamine metabolism pathway include polyamine synthesis metabolites (Put, SAM, SPDS, SPMS) and polyamine decomposition metabolites (PAO, ROS) [29]. Therefore, the increase in Spm and the unchanged Spd on the 18 d in this study are due to multiple primers regulating their expression levels. At 18 d, the content of Spm in callus cultured with D-Arg was significantly higher than that in the control, and the (Spd + Spm)/Put ratio increased. This is consistent with the results with longan as material, in which a high ratio of (Spd + Spm)/(Put) promotes somatic embryo development [30].

As PA metabolism is transcriptionally regulated, we analyzed the expression of genes related to PA metabolism in D-Arg treated media. According to our results, compared with the control, exogenous D-Arg significantly reduced the activity of ADC in EC; however, the expression of *LITCHI001935.m1* (ADC family) was significantly increased (Figure 2, Figure 3 and Figure 8). The expression pattern of ODC and DAO enzyme activity and related gene expression were similar to ADC. We propose that exogenous D-Arg improves the expression level of these gene families, which is due to the stress response mechanism caused by the reduction of the content of Put by exogenous D-Arg. This is the same as the results of the study on the effects of exogenous PAs on the development of longan SE [2]. At 9 d, the expression of *LITCHI017380.m1* (SPDS/SPMS family), *LITCHI017380.m2* (SPDS/SPMS family), *LITCHI000635.m2* (PAO family), and *LITCHI002066.m1* (PAO family) was significantly downregulated compared to that in the control, further indicating that the reduction of Put led to a decrease in the synthesis and decomposition of PA (Figure 8). The majority of genes in the PA metabolism gene family were upregulated at 18 d, indicating that initiating embryogenesis during SE requires the involvement of a large amount of PA substances (Figure 8).

### 3.2. Exogenous D-Arg Regulates EC Proliferation and Somatic Embryo Induction in Litchi by Altering Endogenous Hormone Levels

Previous studies have shown that the crosstalk between polyamines and hormones plays an important role in plant embryos [31,32]. To provide evidence for the influence of applied D-Arg on the metabolism of endogenous hormones in EC and somatic embryo in litchi, we determined endogenous hormones with the exogenous D-Arg treatment as well as the control group. Exogenous D-Arginine did not cause differential levels of metabolites for endogenous hormones in embryonic callus tissue compared to the control at 9 d. At 18 d, however, exogenous D-Arg caused significant differences between the IAA Gly of auxin class, K9G, DHZ7G, and tZR of cytokinin class, and JA Val, JA, and JA-ILE of jasmonic acid class and the control group (Figure 4).

In most species, auxin, cytokinin, and jasmonic acid are the key factors triggering the embryogenic response, as they have regulatory effects on cell division and differentiation [33,34]. Many studies have shown that there is a crosstalk between auxin and cytokinin metabolism during cell development [35,36]. In our study, exogenous D-Arg increased the IAA-Gly and DHZ7G content and decreased the content of tZR in EC (Figure 4), which is consistent with results in *Dendrobium officinale* [37] and *Arabidopsis thaliana* [27]. A significant increase in the IAA content was reported to accompany early SE induction in different plants, including pineapple guava [38], cotton [39], and Norway spruce [40]. The results indicate that an increase in auxin content is beneficial for inducing somatic embryos. Tokuji found that during the embryo formation of embryogenic cell clumps, CTK regulates the early stage of auxin-induced somatic embryogenesis in carrots [41]. A high ratio of auxin to cytokinin implies the induction of somatic embryos [42,43,44]. All these indicate that auxin and cytokinin synergically regulate SE. Our results also showed that exogenous D-Arg significantly reduced the content of JA-Val, JA, and JA-ILE at 18 d of inhibition (Figure 4), which is consistent with former research results [45,46], where an increase in arginine content inhibits the biosynthesis and signal transduction of JA, resulting in a decrease in JA content (Figure 2C). The results of Ahmadi et al. [47] and Białecka and Kępczyński [48] indicated that low levels of JA are beneficial for SE.

### 3.3. Exogenous D-Arg Affects the Expression of Genes Related to SE in Litchi

To investigate the effect of exogenous D-Arg on SE at the transcriptional level, we analyzed the transcriptome data of materials cultured with and without D-Arginine at two key time points. In this study, at 9 d, the number of up-regulated genes was higher than that of down-regulated genes, potentially because D-Arg activated the expression level of genes and promoted the biological process of EC proliferation (Figure 5A). At 18 d, the number of DEGs increased dramatically, indicating the importance of transcription translation in promoting SE (Figure 5A). The number of up-regulated genes was significantly lower than the number of down-regulated genes, which may be due to the reduction of the expression level of genes that inhibited somatic embryo development with the application of exogenous D-Arg, and thus promoted somatic embryo development.

Enrichment analysis of differential gene functions is conducted to identify key biological pathways, thereby revealing the molecular mechanisms of biological processes. In this study, at 9 d, KEGG pathway enrichment analysis revealed that the differentially expressed genes between exogenous D-Arg treatment and the control were significantly enriched in “Amino sugar and nucleotide sugar metabolism”, “Alanine, aspartate, and glutamate metabolism”, and “Biosynthesis of nucleotide sugars” (Figure 6A). These pathways provide the material basis and energy for cell division. The biological processes significantly enriched in GO include “cell wall organization or biogenesis” and “cell wall macromolecule metabolic process” (Figure 6C). These results indicate that exogenous D-Arg regulates EC proliferation by regulating litchi cell division. At 18 d, KEGG pathway enrichment analysis showed that the differentially expressed genes of the M3_18 d vs. Ar3_18 d group were significantly enriched in the “ribosome” (Figure 6B). Most of the differentially expressed genes were significantly enriched in “intracellular anatomy”, “intracellular organelles”, “organelles”, “gene expression”, and “cellular nitrogen compound biosynthesis process” in the GO functional enrichment (Figure 6D); the results showed that they had high translation activity at the beginning of somatic embryo, contributing to cell homeostasis in early somatic embryo.

Studies on SE development have shown that transcription factors play an important role in cell differentiation, the ability to maintain embryogenesis, meristem maintenance, and stress and hormone-mediated signal transduction [49,50]. In this study, we identified 35 differentially expressed TFs in the comparisons of the M3_9 d vs. Ar3_9 d group, among them, WRKY, AP2/ERF, C2H2, and C3H, were highly represented. In the comparison of the M3_18 d vs. Ar3_18 d group, 34 differentially expressed TFs were found, among which C3H, WRKY, AP2/ERF, and C2H2 were highly representative. Previous studies have shown that the WRKY [13,51], AP2/ERF [52,53], C3H [54], and C2H2 [55,56] TF families are involved in EC formation and early somatic embryo development. In this experiment, EC with exogenous D-Arg showed different expression patterns during culture. At 9 d, most genes of these gene families were significantly up-regulated, while at 18 d, most of these transcription factor genes were significantly down-regulated. These results showed that D-Arg is involved in EC proliferation and somatic embryo induction by regulating the expression of WRKY, AP2/ERF, C2H2, and C3H TF gene families.

## 4. Materials and Methods

### 4.1. Plant Materials and Treatments

The ‘Feizixiao’ litchi EC used in this study was induced by our research group with ‘Feizixiao’ litchi anther; the preservation of ‘Feizixiao’ EC was subcultured by subculture using M2 medium and M3 medium, according to Wang et al. [14]. After cultured on M2 medium (MS medium, 30 g·L^−1^ sucrose, 2 mg·L^−1^ 2,4-Dichlorophenoxyacetic acid, 1 mg·L^−1^ kinetin, 5 mg·L^−1^ AgNO_3_ and 7 g·L^−1^ agar, pH 5.8) for 20 days, EC was transferred onto M3 medium (MS medium, 30 g·L^−1^ sucrose, 1 mg·L^−1^ 2,4-Dichlorophenoxyacetic acid and 7 g·L^−1^ agar, pH 5.8, control) and Ar3 medium (M3 + 0.35 g·L^−1^ (2 mmol·L^−1^) D-Arg, pH 5.8, treatment). The first day of inoculation was recorded as 0 d. The ECs on the two mediums were weighed on 0 d (G0), 9 d (G9), and 18 d (G18) after inoculation. The reason why we chose stages 9 d and 18 d is that our previous studies have shown that there is a significant difference in callus progenesis on Ar3 and M3 medium at these two stages. The proliferation rate of EC was calculated as (Gtk − G0)/G0 × 100%, (t = 9 or 18, k = M3 or Ar3).

After subcultured on Ar3 and M3 for 18 d, ECs were transferred to T3 medium (MS medium, 60 g·L^−1^ sucrose, 0.1 mg·L^−1^ 1-naphthylacetic acid, 5 mg·L^−1^ kinetin, 0.1 g·L^−1^ inositol, 0.4 g·L^−1^ lactoalbumin hydrolysate, 100 mL·L^−1^ coconut milk and 10 g·L^−1^ agar, pH 5.8) to induce somatic embryos. Somatic embryo induction rate was measured after culture in 25 ± 1 °C continuous darkness for 7 weeks.

Somatic embryo induction rate = number of somatic embryos/initial inoculation amount × 100%

The initial inoculation amount of SE-induced culture was 0.25 gFW·5 utensils^−1^. Each medium was inoculated with 20 petri dishes and the experiment was repeated 3 times.

EC at 0 d, 9 d, and 18 d stages on both M3 and Ar3 medium were sampled and frozen in liquid nitrogen and then stored in a −80 °C refrigerator for later use.

### 4.2. Determination of PAs

The content of PAs was detected by liquid chromatography–tandem mass spectrometry (LC-MS), described by Wang et al. [14]. Briefly, PAs were extracted from 0.2 g EC and homogenized with 10 volumes of 10% (φ) cold acetonitrile. Following centrifugation at 10,000× *g* for 10 min at 4 °C, the supernatants were collected and filtered using a 0.22 µm filter. The standard was treated in the same way as the sample and then put into the instrument (Thermo Scientific/Exactive Plus, MA, USA) for determination.

Conditions of liquid chromatography were: the chromatographic column was Poroshell 120 SB-Aq (2.7 μm, 3.0 × 150 mm); the elution procedure was as follows: mobile phase A: 0.1% formic acid, mobile phase B: acetonitrile (containing 0.1% formic acid); isocratic elution: 90% A, injection volume 2 μL, column temperature 30 °C and flow rate 0.3 mL·min^−1^.

Mass spectrometry conditions: ion source-electrospray ion source; scanning mode-full MS/AIF scanning in positive ion mode; collision energy—35 eV; spray voltage—3.7 kV; the temperature of ion transport tube was—320 °C; auxiliary gas heating temperature—320 °C; sheath gas flow rate—40 arb; auxiliary gas flow rate—15 arb; purge gas flow rate—5 arb; sheath gas, auxiliary gas, and purge gas were all high-purity nitrogen.

After the measurement was completed, the standard curve linear regression equation was calculated using the concentration of the standard as the independent variable and the integrated peak area of each standard as the dependent variable. Substituted the integrated peak area of the sample into the standard curve linear regression equation, and then substituted it into the following equation to calculate the actual content of the substance:

Content (ng·gFW^−1^) = (c × V)/m

Note: c: the concentration value (ng·mL^−1^) obtained by substituting the integrated peak area value in the sample into the standard curve equation, V: volume of extraction solution (mL), and m: sample mass (gFW).

The calculation method for (Spd + Spm)/Put was to first calculate the (Spd + Spm)/Put of each biological replicate sample, and then calculate the average and standard deviation of the samples from three biological replicates.

### 4.3. Determination of Key Enzyme Activity for Polyamine Synthesis and Metabolism

The enzyme activity of ADC, ODC, DAO, and PAO were separately tested. Three biological replicates were measured for each sample. In each replicate, 0.2 g sample was placed in a mortar and then ground by adding 4 mL of phosphate-buffered saline (PBS) buffer until homogenized, and the supernatant was taken after centrifugation at 1500× *g* for 10 min at 4 °C. Then the enzyme activities were determined by plant enzyme-linked immunoassay kit (FANKKEWEI, Shanghai, China). Blank wells were zeroed and the absorbance of each well was measured sequentially at 450 nm wavelength.

After the measurement was completed, the standard curve linear regression equation was calculated using the concentration of the standard substance as the independent variable and the OD (optical density) values corresponding to each concentration as the dependent variable. The OD value of the sample was substituted into the standard curve linear regression equation, and then into the following formula to calculate the enzyme activity:

enzyme activity (U·gFW^−1^) = (c × V)/(1000 × m)

The calculation formula has been converted between units. c: the concentration value (U·L^−1^) obtained by substituting the sample OD value into the standard curve linear regression equation, V: the volume of extraction solution (mL), and m: weighed sample mass (gFW).

### 4.4. Determination of Hormone Levels

Approximately 1.0 g of each sample was rapidly frozen in liquid nitrogen and homogenized into powder. The treated samples were tested for phytohormones by Wuhan Metware Biotechnology Co., Ltd. (Wuhan, China). The quantification of hormones was conducted using AB QTRAP^®^ 6500+ LC-MS/MS system, analytical method using external standard and isotopic internal standard. Auxin, cytokinin, abscisic acid, gibberellins, ethylene, jasmonic acid, salicylic acid, and strigolactone were detected.

With the concentration ratio of external standard to internal standard as independent variable and the peak area ratio of external standard to internal standard corresponding to each concentration ratio as dependent variable, the linear regression equations of standard curves for different substances were calculated. The ratio of the integrated peak area of the sample was calculated by substituting it into the linear equation of the standard curve and then by substituting it into the following formula, the content data of the substance in the actual sample was finally obtained:

The content of hormones (ng·gFW^−1^) = (c × V)/(1000 × m)

Note: the calculation formula has been converted between units; c: the concentration value (ng·mL ^−1^) obtained by substituting the ratio of the integrated peak area into the standard curve in the sample, V: the volume of the solution used in redissolution (μL), and m: Sample mass (gFW).

### 4.5. RNA-Seq Transcriptomics Sequence and Analysis

For transcriptome analysis, three biological replicates of five samples were used. Total RNA was extracted using an RNAprep pure plant plus kit (TIANGEN, Beijing, China) according to the manufacturer’s instructions. RNA quality was checked by electrophoresis on 1% agarose gel and spectrophotometry with an Agilent 2100 bioanalyzer (America). The transcriptome analysis was performed in Beijing Novogene Technology Co., Ltd. (Beijing, China, http://www.novogene.com/ accessed on 8 June 2022). Library construction was carried out according to Illumina standard instructions and was sequenced on an Illumina NovaSeq 6000 platform. Quickly and accurately compared high-quality clean reads with the reference genome using HISAT2 software (Version: HISAT2 2.2.1) to obtain the localization information of reads on the reference genome. TPM (transcripts per million) was used as an indicator to measure transcript or gene expression levels, and the threshold standard of DEGs was |log2Fold Change| ≥ 1 and padj < 0.05. Then, DEGs were mapped to gene ontology (GO) terms in the database (http://www.geneontology.org/ accessed on 10 September 2022), where gene numbers for each term were calculated to identify significantly enriched GO terms based on a hypergeometric test. In addition, pathway enrichment analysis was conducted utilizing the Kyoto Encyclopedia of Genes and Genomes (KEGG, http://www.kegg.jp/ accessed on 10 September 2022) and MapMan (version 3.6.0, http://mapman.gabipd.org/ accessed on 10 September 2022) database. Using interprescan software (Version: InterProScan 5.67-99.0) for superfamily and Pfam annotation, obtained the ID of each gene’s superfamily and Pfam, then using plant transcription factor database (PlantTFDB, http://planttfdb.gao-lab.org/ accessed on 1 November 2022) for prediction of the annotated superfamily and Pfam transcription factor family.

### 4.6. Identification of Genes Involved in PA Metabolism in Litchi

The genes involved in PA metabolism in *Arabidopsis* were obtained from TAIR (http://www.arabido-psis.org/ accessed on 23 March 2023), and the genes involved in PA metabolism in tomato were obtained from EnsemblPlants (https://plants.ensembl.org/Solanum_lycopersicum/Info/Index accessed on 24 March 2023). These genes were used to search the Pfam database (http://pfam-legacy.xfam.org/ accessed on 29 March 2023) for conserved domain sequences of each gene family in polyamine metabolism. Then, the conserved domain sequence and litchi genome sequence were used as base alignment sequences with the local blast by MobaXterm. Genome data for litchi were downloaded from SapBase (http://www.sapindaceae.com/ accessed on 31 March 2023). All of the sequences identified for PA metabolism genes in litchi were subjected to SMART (https://smart.embl.de/ accessed on 3 April 2023), the NCBI’s Batch CD-search (https://www.ncbi.nlm.nih.gov/Structure/bwrpsb/bwrpsb.cgi accessed on 3 April 2023) tools to verify their reliability as targets.

### 4.7. RNA Extraction and Reverse Quantitative Transcriptase-Polymerase Chain Reaction (qRT-PCR)

Total RNA was isolated from the leaf using an RNA isolate kit (Hua Yueyang, Beijing, China). The first-strand cDNA was synthesized with a RevertAid First Strand cDNA Synthesis Kit (Thermo Scientific, Shanghai, China) for quantitative real-time PCR (qPT-PCR). The Primer3Plus (https://www.primer3plus.com/ accessed on 17 July 2023) was used to design the specific primers of the selected genes (Table 2) and the qRT-PCR was performed using TB Green Premix Ex Taq II Kit (TaKaRa, Kyoto, Japan). The reaction system was 10 µL, including 5.2 µL TB Green Premix Ex Taq II, 3 µL ddH_2_O, 1 µL cDNA template, and 0.4 µL forward and reverse primers, respectively. The reaction procedure was as follows: 95 °C for 3 min; 95 °C for 10 s; and 60 °C for 30 s, running for a total of 39 cycles. The expression quantities of genes were calculated according to the 2^−ΔΔCT^ method. Real-time PCR analysis was performed with three biological replications, and each biological replication was made in three technical replicates.

### 4.8. Statistical Analysis

Analysis of the data was performed using SPSS 24.0 (SPSS Inc., Chicago, IL, USA) and Origin 2021 (Origin Lab, Hampton, MA, USA) for analysis of variance and significance. All data are presented as the mean ± standard deviation (SD) of three replicates. Different letters in the significance analysis indicate significant differences between treatments (*p* < 0.05, Duncan).

## 5. Conclusions

This study revealed an intricate mechanism that regulates the proliferation of EC and induction of somatic embryos in litchi. The comprehensive analyses of transcriptomic and physiological data revealed that exogenous D-Arginine synergistically regulates litchi EC proliferation and somatic embryo induction in litchi through polyamine metabolic pathways, endogenous hormone levels, and SE-related TFs. In conclusion, this study provides valuable information on the underlying molecular and physiological mechanisms during the SE process of litchi.

## Figures and Tables

**Table 1:** Identification of polyamine metabolism genes in the litchi genome. The genes marked gray were not expressed in the EC of litchi.

Family	Pfam	Description	Gene ID
ODC	PF00728	C-terminal sheet domain	*LITCHI001849.m1*
	PF02784	Pyridoxal-dependent decarboxylase, pyridoxal binding domain	*LITCHI010045.m1*
ADC	PF02784	Pyridoxal-dependent decarboxylase, pyridoxal binding domain	*LITCHI001935.m1*
SAMDC	PF01536	Adenosylmethionine decarboxylase	*LITCHI014637.m1*
			*LITCHI005415.m1*
			*LITCHI019298.m1*
SPDS/SPMS	PF01564	Spermine/spermidine synthase domain	*LITCHI014771.m5*
	PF17284	Spermidine synthase tetramerisation domain	*LITCHI014771.m1*
			*LITCHI017380.m2*
			*LITCHI017380.m1*
			*LITCHI009625.m1*
			*LITCHI009625.m3*
			*LITCHI022355.m1*
			*LITCHI004977.m1*
DAO	PF01179	Copper amine oxidase, enzyme domain	*LITCHI014772.m1*
	PF02727	Copper amine oxidase, N2 domain	*LITCHI014780.m1*
	PF02728	Copper amine oxidase, N3 domain	*LITCHI014775.m1*
			*LITCHI014781.m1*
			*LITCHI022471.m2*
			*LITCHI022850.m1*
			*LITCHI004876.m1*
PAO	PF01593	Flavin containing amine oxidoreductase	*LITCHI015688.m7*
			*LITCHI015688.m1*
			*LITCHI017627.m1*
			*LITCHI013235.m1*
			*LITCHI026697.m1*
			*LITCHI027276.m2*
			*LITCHI027276.m5*
			*LITCHI001171.m1*
			*LITCHI000635.m2*
			*LITCHI000635.m1*
			*LITCHI002066.m1*
			*LITCHI003677.m1*
			*LITCHI009613.m1*
			*LITCHI008116.m1*
			*LITCHI008116.m2*
			*LITCHI010016.m1*
			*LITCHI010183.m1*
			*LITCHI022368.m1*
			*LITCHI020715.m1*
			*LITCHI020181.m4*
			*LITCHI020181.m1*
			*LITCHI004421.m1*
			*LITCHI019019.m1*

**Table 2:** Primer sequences and produce size in qRT-PCR analysis.

Gene Name	Primer Sequences (5′ to 3′)		Produce Size (bp)
	F	R	
*LITCHI009273.m1*	AGTAAGGGAGCCGAACAAGAAGTC	CAAGCCGAGTCAGCAAAGTTGAG	138
*LITCHI011747.m2*	TGCCAACAAGTCGCCGAAGG	TCCAAGTCCAACTACTGCTGCTC	107
*LITCHI013074.m1*	ATTCCAACGAGATCCGATACAGAGG	CGAAGGTTCCGAGCCAGACAC	108
*LITCHI014933.m1*	AGATGGTGACTGGATGCTGGTTG	GCCTCTTGCTTCTGTTCCTTTCATG	97
*LITCHI022471.m2*	GCTGCTGCTGTCACTCTTCTTG	GGTTGGATGAGGGAGGCGATAG	89
*LITCHI022746.m1*	AGCGGCGATTTGGGACTTGG	AACGGCGGATGAGGCGATAAC	90
*LITCHI027327.m1*	CACCATCACCACCAACAGCAAAC	TCTCACCAAGGAATTAACCCAGGAC	83
*LITCHI001849.m1*	CTGACAACCTCACCACCAACAC	CTCGCAGTAAAGGAAGCCATCG	74
*LITCHI001935.m1*	GGAGTGGTGGTGATTCTGATGATG	ACACGATGACGGCACAACAAG	137
*LITCHI019298.m1*	TTGCTAGGGCTGATGGCTCTG	ACTTCCTCGTCCTTCTCGTCTTC	79
*LITCHI004977.m1*	AGGTTCTGGTTATTGGTGGAGGAG	AAAGCCCACAGCCACACTAGG	146
*LITCHI022850.m1*	GCTTCCTCTGCCTTACCATCGG	GTTTAGCGTGGAAAGGGTTCTTGG	150
*LITCHI017627.m1*	GGCAGGAATGGCTGGTCTCAC	TGACCCACCTTCCACAACACATAG	94
*β-actin*	TTGGATTCTGGTGATGGTGTG	CAGCAAGGTCCAACCGAAG	80

## Data Availability

Data are contained within the article and Supplementary Materials.

## Supplementary Materials

The following supporting information can be downloaded at: https://www.mdpi.com/article/10.3390/ijms25073965/s1.
